# Lower Intrafamilial Transmission Rate of Hepatitis B in Patients With Hepatitis D Coinfection: A Data-Mining Approach

**DOI:** 10.5812/hepatmon.7652

**Published:** 2013-05-23

**Authors:** Omid Pournik, Seyed Moayed Alavian, Leila Ghalichi, Bashir Hajibeigi, Amir Reza Razavi, Saeid Eslami

**Affiliations:** 1 Department of Medical Informatics, Faculty of Medicine, Mashhad University of Medical Sciences, Mashhad, IR Iran; 2 Middle East Liver Disease Center, Tehran, IR Iran; 3 Deputy for research, Iran University of Medical Sciences, Tehran, IR Iran; 4 Iranian Blood Transfusion Organization Research Center, Tehran, IR Iran; 5 Medical Informatics Research Center, Department of Medical Informatics, School of Medicine, Mashhad University of Medical Sciences, Mashhad, IR Iran

**Keywords:** Data Mining, Hepatitis B Virus, Hepatitis D, Machine Learning

## Abstract

**Background:**

The presence of an infected family member significantly increases the risk of HBV transmission, but many socio-demographic and viral characteristics of family members affect the transmission rate.

**Objectives:**

In this study, we have used data mining techniques to investigate the impact of different variables in intrafamilial transmission of HBV infection.

**Patients and Methods:**

demographic information, viral markers, and medical history of 330 patients with chronic hepatitis B and their offspring attending a referral center in Tehran were collected. Data-mining techniques were administered to detect patterns.

**Results:**

The overall transmission rate was 15.7% (5.4% and 27.3% for male and female index cases respectively). In female patients, HBe Ag positively affected the transmission rate (49% vs. 23.4%). There was a dominant change in transmission rate of female patients with negative results for Hbe Ag with HDV coinfection, where the transmission rate changed from 25% in patients with negative results for HDV Ab to 5% in those with positive results. In Hbe Ag negative male index cases, the transmission rate was 1.3% in cases with positive results for HDV Ab compared to 7% in those with negative findings. The overall transmission rate was statistically different between patients with positive and negative results for HDV Ab (P = 0.016).

**Conclusions:**

There is a minor but consistent pattern change in the presence of HDV infection which reduces familial transmission of HBV, especially in female patients with negative results for HBe Ag.

## 1. Background

The prevalence of Hepatitis B virus (HBV) infection varies among the countries around the world (1). Chronic hepatitis B infection affects less than 0.5% to more than 10% of people in different parts of the world, and it is estimated that more than 350 million people are infected worldwide (2). It is a major public health issue and has a high burden on health systems (3). Apart from acute manifestations, the infection imposes high burden on health systems especially due to its sequels; cancer and cirrhosis. One of the determining factors associated with high burden of hepatitis B is the relative high transmission rate of the infection. The intrafamilial transmission of the infection is an important concern for patients and their family as well as health service providers and policy makers. Many strategies such as neonatal vaccination and hepatitis B immunoglobulin (HBIG) injection to the newborns of infected mothers have been proposed and administered (4). The disease can be transmitted both vertically (from infected mothers to offspring) and horizontally (between the spouses or children) in the family (5). Both perinatal transmission from mother to child and horizontal transmission from parents and other sibling during childhood are important (6).It is estimated that about 90% of vertically infected infants would progress to chronic hepatitis. About 30% to 50% of the infected children between the first and 5th year will become chronically infected while the rate is as low as 5% in adults (7). Acquiring the disease in younger age increases the chance of developing chronic disease significantly, which highlights the importance of protective measures against newborn infection (2). Many studies have shown that the presence of an infected family member significantly increases the risk of HBV transmission (5, 8) while the risk of transmission varies based on socio-demographic variables, family role (mothers infection increases the risk of transmission) and viral markers of the index case (5, 6, 9) .Iran has low to intermediate HBV prevalence (3) .The prevalence has declined in recent years probably due to national vaccination programs (3).

Vaccination against HBV has changed the global picture of HBV infection, but still a considerable number of people become infected annually. The coverage of vaccine is not complete, and many high risk populations are not vaccinated (2) .Even in vaccinated populations, the long term protective effect of the HBV vaccine is not clear, and immunity may diminish after a few decades or when immune system is weakened due to other health conditions (10) or when some of the HBV subtypes are present (11). Discovering hidden patterns in transmission of Hepatitis B virus can be a start point in developing techniques to reduce transmission of the infection. The development of information systems and medical databases has provided the opportunity to use machine learning techniques to detect the associations and patterns between numerous variables in medical fields. These methods are effective in investigating medical field data with high level of uncertainty, missing and inaccuracy (12). Data mining techniques can extract unknown and useful information and pattern about the data. Policy makers and healthcare providers can benefit from the extracted patterns, to improve the quality of care, reduce the costs, and forecast the need for resources and prognosis of the patients (13). In some cases the extracted patterns can be applied even without full comprehension of the casual mechanisms (14). Investigating the transmission patterns in infectious disease can result in discovering new patterns and identifying methods to reduce transmission and better allocating resources (15).

## 2. Objectives

In this cross-sectional study, we aimed to investigate the intrafamilial transmission patterns in families with HBV infection and explore the variables which could change the patterns and transmission rates between the subgroups.

## 3. Patients and Methods

In this cross-sectional study, demographic information, viral markers, stage of disease (inactive carriers, chronic hepatitis and liver cirrhosis), and family history of liver disease of all cases with chronic B hepatitis referred to Middle East Liver Disease (MELD) center were gathered between 2009 and 2011. This is a referral center in the city of Tehran which admits referred patients from other clinics in the city as well as all over the country. The index case was the first parent in the family who was identified as HBV infected. Demographic information and viral markers of their offspring were also collected. The participants were cases with chronic HBV infection (more than six months with positive findings for HBs Ag). Patients with HIV, HCV, acute hepatitis B infection, and patients never married, younger than 18 years old, and those who did not provide their written consent were excluded from the study. All data were recorded by one physician in a check list based on medical records, physical examination, and past medical history of the patient and his or her offspring. The final diagnosis was made by a specialist. All patients provided their written informed consent before the data collection. The data were analyzed using network analysis method where each case was evaluated in respect to all other cases. This method is applied to explore the structural and relational aspects of various health issues such as disease transmission (e.g. HIV/AIDS and other sexually transmitted diseases), information transmission, and health aspects of social networking (16). We used feature selection analysis to choose the effective factors and include them in the model. Feature selection which is also called variable selection or feature reduction has been used to select the most relevant variables, and to help removing irrelevant and redundant ones from learning process of modeling. This phase has a paramount importance for the next step which is extracting best and most reliable patterns from data. The importance of this step is due to the fact that entering poor set of features (incomplete, noisy, irrelevant and redundant) may result in difficulties in finding patterns. It may even end in identifying poor and misleading patterns. At the other hand feature selection helps to identify the most and least relevant fields for analysis. Feature selection can be used for two aims: first for feature ranking and extracting most important features, and second for subset selection to search for the set of features that can be included in the optimal subset. In this study we used feature selection for feature ranking and no subset selection was performed. There are several methodological approaches available for feature selection, and we used Brute force method which selects the most important attributes by trying all possible combinations of attributes in data set. RapidMiner version 5 was used to perform the analysis. We used CHAID (Chi-squared Automatic Interaction Detection) algorithm for modeling, which is a decision tree representation model, based upon adjusted significance testing of Bonferroni. CHAID algorithm evaluates the associations between variables by exploratory analysis methods, and is an enhanced alternative to multiple linear regression and logistic regression. The original CHAID algorithm was introduced by Kass for nominal dependent variables, and extended to ordinal dependent variables by Magidson (17). CHAID output is highly visual and easy to understand like other decision tree representing algorithms. The main advantage of CHAID is its ability to build nonbinary tree (trees with more than two branches attached to a node). Statistical analysis was performed using SPSS version 16. Data were presented as mean and standard deviation, and Chi square and fisher exact test were used for analysis of categorical data.

## 4. Results

**Figure 1.**
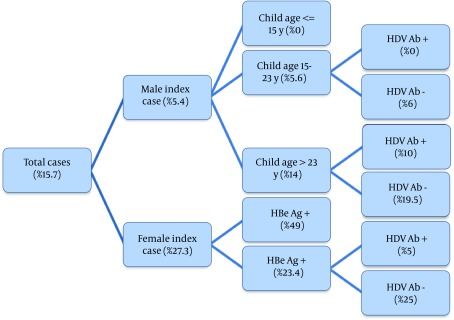
The Transmission Rate (%) of Patients in Different Subgroups of Patients, the Variables and Their Sequence Are Derived from the Model

A total of 390 patients, 176 (45%) females and 214 (55%) male participated in this study. Mean age of the participants was 47 years (SD: 11.7), and they were between 18 and 71 years old. Patients were referred to clinic following screening or routine check-up (33%), positive findings during blood donation (27%), clinical symptoms (21%), and having a case with positive results in their family (19%). Fifty one patients had positive results for HBe-Ag, and 41 patients had positive findings for HDV-Ab. The participants had 909 offspring (43% female), from whom the viral markers of 740 (48% female) were available. The age range of the offspring was between 1 and 45, and the mean age was 17 (SD: 9.9). The final model can be observed in Figure 1. Gender, age of children, HBe Ag and HDV Ab were present in the model. The overall transmission rate was 15.7% (95% confidence interval was 13% – 18.5%). Gender of the index case was the first effective variable. The most important finding in this model was the difference in transmission pattern for male and female patients (Figure 1). The transmission rate was 5.4% when index case was male, and 27.3% when index case was female (P < 0.001). In female patients, HBe Ag had a determining effect on transmission rate (49% in mothers with positive results for HBe Ag compared to 23.4% in mothers with negative results for HBe Ag, P < 0.001). There was a dominant change in transmission rate of female patients with negative results for HBe Ag with HDV coinfection, where the transmission rate changed from 25% in patients with negative results for HDV Ab to 5% in those with positive results. There was only one female index case with both HBe Ag and HDV Ab positive results, and the transmission rate was as high as 49%. In male patients, age of children was associated with different transmission rates (higher rate in older children). Male index cases had no infected child younger than 15 years old. HDV coinfection also had a similar effect on transmission pattern of male index cases, and it reduced the transmission rate from 19.5% to 10% in children older than 23and from 6% to 0% in children between 15 and 23 years old. There was no male index case with positive results for HBe Ag and HDV Ab. For HBe Ag negative male index cases, the transmission rate was 1.3% in cases with positive results for HDV Ab compared to 7% in those with negative findings (P = 0.01). The transmission rate was 2.4% (95% confidence interval was 1%-12.8%) for HDV Ab positive index cases, and 16.4% (13.7%-19.4%) for HDV Ab negative index cases. The prevalence was significantly different (P = 0.013).

## 5. Discussion

In this study we have proposed a decision tree for familial transmission of Hepatitis B which can help explore the disease transmission in patients’ offspring. The difference in decision tree in men and women is a primary finding of this study, which is observed in the previous studies (5, 6, 18). The difference is not observed in some studies, although the transmission rate was higher when both parents were involved (8). The higher maternal transmission rate might be due to perinatal transmission of HBV, which can be reduced through preventive measures. Researches have shown that the introduction of HBV vaccination has resulted in lower intrafamilial transmission of HBV (19). Researchers have suggested screening pregnant women for HBV infection to reduce perinatal transmission (20). The transmission may be present even after vaccination, especially in neonates of mothers with positive results for HBeAg. HBIG injection is also proposed to reduce perinatal transmission of the infection; although, it cannot fully protect the neonate (21). In male patients, age of children is an important variable which can be considered as a proxy of duration of exposure. No case of transmission was observed in children younger than 15 years. In Iran, national vaccination of newborns against HBV started since 1993 which might have resulted in the reduced paternal transmission in this age category. In female patients who have higher transmission rate, it is the presence of HBeAg which determines transmission rate, similar to findings of Salkic et al. (22) but not confirmed in some other studies (23, 24). Age of the offspring did not change the transmission rate in contrast to male index cases. In our study, HDV infection decreased the transmission rate of HBV between family members. There is a consistent pattern change in the presence of HDV infection which reduces familial transmission, especially in patients with negative findings for Hbe Ag. Introduction of safe and effective HBV vaccines and integration of HBV vaccination in national immunization programs of many countries have resulted in decreased Hepatitis B infection, which in turn reduced HDV infection despite its high infectivity (25, 26). Xiridou et al. have introduced a mathematical model which shows that hepatitis D infection modulates both the severity of the HBV epidemic and the impact of interventions for HBV (27). The frequency of HDV-HBV coinfection should be taken into account when investigating such effects. The aforementioned rate is 7.8% in Iran which is lower than endemic countries (28). It is possible that the coinfection of HBV and HDV may reduce the viral load or weaken transmission by some other mechanism. This is a cross-sectional study and its ability to explore the causal associations is limited. The applied data mining methods is aimed to detect patterns and associations. Further studies are required to confirm these exploratory findings, and evaluate the consistency of these findings in different populations. If confirmed, effort can be made to place these patterns in hepatitis B transmission predicting models. Although vaccination is an effective protective method for hepatitis B infection, the induced immunity may diminish through decades (11). Other protective methods should be discovered and introduced to improve global efforts and reduce burden of the disease. The observed protective effect of HDV infection can be a sign of an underlying mechanism. Other investigations in this field may result in a better understanding of the pathogenesis of the disease which in turn may lead to introduce new protective methods.
